# An Efficient Method to Identify Conditionally Activated Transcription Factors and their Corresponding Signal Transduction Pathway Segments

**DOI:** 10.4137/bbi.s3485

**Published:** 2009-11-17

**Authors:** Haiyan Hu

**Affiliations:** School of Electrical Engineering and Computer Science, University of Central Florida, Orlando, Florida, USA. Email: haihu@cs.ucf.edu

**Keywords:** signal transduction pathway, transcription factor, microarray gene expression, conditionally activated pathway

## Abstract

A signal transduction pathway (STP) is a cascade composed of a series of signal transferring steps, which often activate one or more transcription factors (TFs) to control the transcription of target genes. Understanding signaling pathways is important to our understanding of the molecular mechanisms of disease. Many condition-annotated pathways have been deposited in public databases. However, condition-annotated pathways are far from complete, considering the large number of possible conditions. Computational methods to assist in the identification of conditionally activated pathways are greatly needed. In this paper, we propose an efficient method to identify conditionally activated pathway segments starting from the identification of conditionally activated TFs, by incorporating protein-DNA binding data, gene expression data and protein interaction data. Applying our methods on several microarray datasets, we have discovered many significantly activated TFs and their corresponding pathway segments, which are supported by evidence in the literature.

**Availability:** http://www.cs.ucf.edu/~haihu/Download/BBI/ACTPATH.htm

## 1. Introduction

Signal transduction pathways (STPs) reflect complex biological processes during which the cell converts one signal to another. The activation of STPs often activates downstream transcription factors (TFs), which then bind to their target genes to turn on or off various transcription programs. Understanding STPs will significantly augment our understanding of specific cellular mechanisms.

STP activation is condition-dependent. A number of curated condition-annotated STPs have been deposited in various databases.^1^ However, considering the large number of ligands and receptors, and the many possible conditions, the STPs collected in current pathway repositories are far from complete. Novel computational methods have been developed to model STPs as chains or networks of interacting proteins and to identify STPs by assuming that genes in the same pathway are more likely to have correlated microarray expression. 2–6 However, genes in the same STP may not have well-correlated gene expression. Additionally, current methods for STP identification seldom consider the condition dependence of STPs. Even though intracellular signal transductions ultimately effect transcriptional changes and the activation of a pathway downstream TF often indicates the phenotype-relevance of its corresponding STP, current methods rarely investigate STP-corresponding transcription regulation programs during STP modeling.

In this paper, we propose an efficient method to identify differentially activated TFs and their corresponding pathway segments by incorporating DNA-protein binding data, protein interaction (PPI) data and microarray expression data. We first design a statistical method to identify differentially activated TFs. We then design a graph algorithm ACTPATH to identify pathway segments corresponding to each identified differentially activated TF. The ACTPATH algorithm applies a random walk method to the PPI network to discover significant protein interactions. By identifying connected PPI subnetworks from significant protein interactions, the algorithm will output potential pathway segments corresponding to the identified activated TFs. As an experimental study, we applied our approach to breast cancer and essential thrombocythemia (ET) microarray data sets and identified dozens of TFs differentially activated under given conditions. We also predicted a number of TF-corresponding pathway segments. Statistical assessment and a literature search demonstrate the efficacy of our approach.

## 2. Method

As we mentioned before, the activation of TFs is one good indicator of activation of their corresponding pathways. Based on this observation, we start by identifying activated TFs from a two-condition microarray experimental dataset (Section 2.1). We then describe the algorithm ACTPATH, which identifies TF-corresponding pathway segments (Section 2.2).

### 2.1. Identification of differentially activated TFs

TFs are often regulated at the post-transcription level, and thus it is often hard to identify TF activity by directly measuring the change in expression of their corresponding mRNAs. Therefore, we will determine differentially activated TFs by expression of its target genes. Our assumption is that if the target genes of a TF are differentially expressed, it is most likely that the TF is differentially activated. Note that a TF could bind to different target genes under different conditions. The target genes a TF binds to under a given condition are called condition-specific target genes for this TF.

If we assume target genes of a differentially activated TF are more likely to be differentially expressed than those of an inactivated TF, and we also assume the condition-specific target genes of an activated TF are known, then the identification of activated TFs in two-sample comparison microarray experiments could be done simply by using a hypergeometric test described as follows. Assume there are *N* genes on the microarrays and n of them are differentially expressed. For one TF, assume we know there are *M* potential target genes on the arrays and m of them are among the n differentially expressed genes. Then the following hypergeometric test will assess the overrepresentation of the TF target genes in the differentially expressed gene list by a p-value of $\sum_{i=m}^{\text{min}(n,M)}\frac{C_{M}^{i}C_{N-M}^{n-i}}{C_{N}^{n}}$.

However, it is difficult to simply apply the hypergeometric test because we do not know which genes are differentially expressed without applying some arbitrary cutoff for the differential test statistic. Zilberstein et al proposed the minimal hypergeometric test (mHG) to avoid the arbitrary cutoff when defining the differentially expressed genes.^8^ The basic idea of the mHG is to try all possible cutoffs to define differentially expressed genes and then choose the cutoff with the smallest hypergeometric p-value and determine the significance of such a cutoff.

However, the mHG is not suitable for our task of identifying differentially active TFs either. There are two reasons for this. One, the mHG does not explicitly take the rank of the genes into account. Given a cutoff, all the genes above the cutoff are treated in the same way. However, the more significantly differentially expressed, the more related a gene is to the given condition. The TFs binding to these more significantly differentially expressed genes are more likely to be conditionally activated. The other is that, the mHG assumes the condition-specific target genes of a TF are known, which is often not the case. Therefore, the mHG cannot be directly applied here to properly select significantly differentially activated TFs.

To identify differentially activated TFs for two-sample comparisons, we propose incorporating two tests measuring different aspects of the conditional relevance of a TF. One is to test whether the m condition-specific target genes rank at very top of the n genes, using a U-test. The other is to test whether there are significantly more condition-specific target genes in the top n genes compared with the rest using hypergeometric test.

Our method to combine the aforementioned two tests includes the four steps outlined below. At step 1, like the mHG, we sort the genes according to the p-value of differential expression. At step 2, for any TF, at any place in the rank list, we calculate a U-test p-value and a hypergeometric p-value. The product of the two p-values will be considered to be the p-value for this TF to be differentially activated, corresponding to this specific cutoff. At step 3, the smallest p-value is picked as the p-value for this TF to be differentially activated. At step 4, we will calculate the false discovery rate (FDR)^9^ and select the most significantly activated TFs. The details of the second and the fourth step are as follows.

Here we detail step 2. For a cutoff in the ranked gene list, we calculate the U-test p-value. Basically, we assume that for an active TF, its target genes should be ranked highest. That is, the higher the target gene’s rank, the more relevant the TF. Assume that, for a special cutoff, we have n differentially expressed genes. For one TF, assume the ranks of its target genes are *x*_1_, *x*_2_, … *x_m_* We want to determine how these m target genes are ranked. The U-test is perfect for such a purpose. It calculates the sum of the ranks of the target genes and compares the sum with that obtained by randomly ranking the n genes. When n and m are large than 10, the normal distribution can be used to approximate the distribution of the sum of the ranks. The mean of the normal distribution is m*(*n* – *m*)/2 and the standard deviation of the normal distribution is $\sqrt{m(n-m)(n+1)/12}$.

With the p-value from the U-test, we further calculate a hypergeometric test p-value. Because the two tests are independent, we use the product of the two p-values as the p-value of the TF activity.

With many TFs having been tested this way, at Step 4 we control the FDR in our prediction of the activated TFs. We will select the active TFs by a q-value calculation described below. First, we rank the TFs according to their p-values, calculated above from the smallest to the largest. Then we calculate the q-value, using the Q-VALUE software.^9^ Third, we identify the largest k such that kq_k_ < *α*. Here the q_k_ is the FDR when we select the k TFs with smallest p-values. The idea is that if kq_k_ is smaller than α, we will make at most α false predictions. We set α as 0.05 in this paper.

### 2.2. Identification of activated STP segments corresponding to activated TFs

With the differentially activated TFs from two-condition comparison microarray data identified above, we design the following algorithm ACTPATH (Algorithm 1), to identify TF-corresponding STP segments from a human protein interaction network.

Two assumptions of the ACTPATH algorithm to identify TF-corresponding STP segments include: 1) in comparison with genes not involved in condition-relevant pathways, those genes that are involved in condition-relevant pathways are more likely to be differentially expressed. This assumption has been made in recent studies on phenotype relevant pathways, such as,^10^ Also, 2) pathway genes should not be too far away from the given TF in the protein interaction network. This assumption is based on several previous studies.^2^,^3^,^11^ Based on the first assumption, we will choose proteins whose genes are more differentially expressed with larger probabilities. Based on the second assumption, we restrict our search of pathways to within a short range around the TF in the protein interaction network.

We model the human protein interaction network as a graph G, in which each node represents a protein, and each edge represents interaction between two proteins. For a given TF identified to be activated, we describe the algorithm as following.

**Algorithm 1** ACTPATH(G,TF)

1. For each node v in G, identify the shortest distance SP(v,TF) between v and the given TF;
2. Construct a subgraph sub(G) containing only nodes within a distance k of TF;
3. Perform a random walk on sub(G) to identify a significant edge set E representing condition-relevant protein interactions with high statistical significance;
4. Construct a subgraph G’ of sub(G) containing only significant edges E;
5. Identify and output connected components from G’ as pathway segments corresponding to the TF.

At steps 1–3 of Algorithm 1, we try to identify pathway edge candidates. We assume that pathway genes should be within a limited range of the given TF in the PPI network. Therefore, at steps 1–2, based on the second assumption, we first extract a subgraph sub(G) from G, containing only nodes within a certain distance to the given TF. At step 3, to identify edges most likely to exist in the pathway corresponding to the given TF, we perform a random walk starting from the TF on the subgraph sub(G). This step is based on the aforementioned first assumption.

The main procedure in step 3 is the random walk. A random walk is a stochastic process generated by a Markov chain. Given the current state of the random walk, say protein *i*, the next state of the random walk will be determined by the transition matrix *P* of the Markov chain. That is, if the random walk currently is at protein *i*, at the next step, the random walk will be at protein *j* with probability *p_ij_*. To define the transition probability, we take the rank of differential expression of each gene into account. Assuming the random walk currently is at protein *i*_0_ and the proteins 1, 2, …, *m* are connected with *i*_0_ in the protein interaction network, and the rank of these m proteins is *r*_1_, *r*_2_, …, *r_m_*, then we define the transition probability from *i*_0_ to the *i*th protein as $e^{-r_{i}/b}/\sum_{j=1}^{m}e^{-r_{i}/b}$. Here *b* is a scale parameter indicating how small the rank should be to claim a gene as differentially expressed. Empirically, we recommend a *b* value of n/2 or smaller if one believes the top 2 × *n* genes are more reliable differentially expressed genes. Besides the general transition rule, we also force the random walk to return to the TF with probability 1 when the random walk arrives at the boundary of sub(G). The boundary is defined as those proteins where the distance between the TF and the protein is equal to a pre-defined threshold. This is to ensure the walk identifies interesting pathways around the TF, instead of wandering around the uninteresting proteins at the boundary.

One can easily prove that the above Markov Chain is irreducible and positive recurrent. According to probability theory,^12^ an irreducible and positive recurrent Markov chain will have a unique stationary distribution π and the transition matrix will converge to the stationary transition matrix $P^{*}=\underset{n\to \infty }{\text{lim}}p^{n}$. Note that there could be thousands of proteins in sub(G). The transition matrix can be huge. Consequently, calculating P* by computing *P^n^* can be expensive. Therefore, instead of calculating P* and π, we run the random walk many times until it converges. To judge whether the random walk converges, we calculate the frequency of visiting each protein after *n* steps of random walking. If the change of the frequency of visiting each protein is smaller than a predefined threshold 0.01, the frequency will be used to approximate π and the transition frequency between two proteins will be used to approximate P*. After the random walk converges, we will output the top 1% of the most visited edges as the significant edges. Such significant edges not only show that the proteins connected by the edges are frequently visited, but also show that the edges themselves are frequently visited.

At steps 4–5, with the significant edges defined, we construct a subgraph G’ of sub(G). G’ only contains significant edges. We will then identify and output connected components from G’ as pathway segments.

## 3. Experimental Study

### 3.1. Data collection

We collected two microarray data sets. The essential thrombocythemia (ET) data set^11^ consists of samples from 16 patients, 9 of 16 of whom have a JAK2 V617F mutation and 7 of 16 of whom do not have a JAK2V617F mutation. The breast cancer data set^12^ consists of samples from 286 patients: 209 of 286 patients are ER positive and 77 of 286 patients are ER negative. For these microarray data sets, we first classified all the samples into two relevant conditions. For example, for the breast cancer data set, we group samples corresponding to ER positive and ER negative respectively. We next used a differential t test to test whether a gene is differentially expressed between the two relevant conditions. The differential test is able to assign to each gene a p-value. All the genes are then ranked according to this p-value. We downloaded TF-target information from the mSigDB database.^13^ This TF target gene data is collected from curated data in the TRANS-FAC database^14^ and/or predicted by comparative genomics approaches.^15^ Note that the downloaded TF target genes are candidate target genes, because a TF may bind with different subsets of its target gene candidates under different conditions. We also downloaded protein interaction information from HPRD.^16^

### 3.2. Identified differentially activated TFs

For the breast cancer dataset, we identified fourteen TFs (Table 1). The involvement of all of these TFs in the disease mechanism of breast cancer is validated by a literature search. For example, Sp1 has been reported to play a key role in basal and estrogen-induced growth and gene expression in breast cancer cells.^17^ Also, Interferon regulatory factor-1 (IRF-1) has been implicated as a tumor suppressor in breast cancer, and is associated with caspase activation and induction of apoptosis.^18^

For ET data, we identified eighteen activated TFs with a q-value cutoff of 0.05 (Table 2). Similarly, we observed evidence in the literature of association between the identified TFs and myeloproliferative disorders. For example, STAT has been documented to have an important role in ET,^19^ and GATA1 has been reported to be associated with myeloproliferative disorders.^20^

### 3.3. Identified STP segments

With differentially activated TFs obtained under the given conditions, we applied our ACTPATH algorithm to identify STP segments corresponding to the activated TFs in the microarray experiments. Because of the lack of a gold standard of activated pathways under any given condition, we will assess the efficacy of our approach by evidence obtained from the existing biological knowledge. We use the PubMatrix tool from NCBI,^21^ which is a web-based application that allows a simple systematic approach to querying the medical literature in PubMed to assign genetic, biological, or clinical relevance to genes of interest.

**Figure 1.**
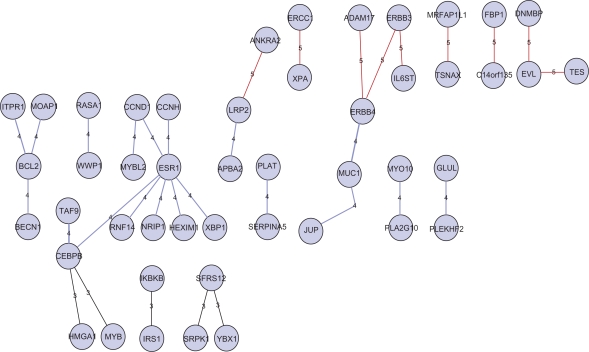
Example of pathway segments corresponding to FALZ identified from breast cancer data (Figure drawn with Cytoscape).^7^

We applied our approach to breast cancer data and found many interesting pathway segments. As an example, Figure 1 shows the pathway segments corresponding to the bromodomain PHD finger transcription factor (FALZ). The relevance matrix generated by PubMatrix (Table 3) corresponds to the pathway segment of JUP-MUC1-ERBB4-ERBB3-ADAM17 in Figure 1. Each entry (*i*,*j*) in this matrix, when *i* is not equal to *j*, stores the number of co-occurrences of the *i*th and *j*th term in literature searched by PubMatrix. We can see from Table 3 that all five genes are relevant to the term “breast cancer”, and JUP is relevant to MUC1, MUC1 is relevant to ERBB4, ERBB3 and ADAM17 and so on. This relevance matrix shows that the five genes in the pathway segments are associated with each other and with breast cancer. Because there may be false positives from PubMatrix results, we also manually searched relevant literature and further validated the biological significance of our predictions. For example, the DF3/MUC1 transmembrane oncoprotein has been shown to be aberrantly overexpressed in most human breast carcinomas, and interacts with the Wnt effector γ-catenin (JUP).^22^,^23^ A novel function of increased MUC1 expression, potentiation of erbB signaling through the activation of mitogenic MAP kinase pathways has been implicated in breast cancer.^24^

**Figure 2.**
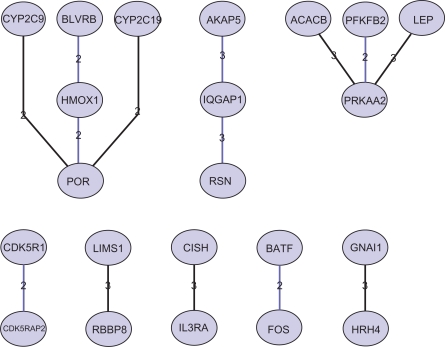
Example of pathway segments corresponding to CREB1 identified from ET data.

Figure 2 shows another example of pathway segments corresponding to cAMP responsive element binding protein 1 (CREB1) identified from ET data. The PubMatrix results indicate significant associations between genes in these identified pathway segments (Table 5). For example, CYP2C19 is relevant to POR and STAT, and POR is further associated with HMOX1, CYP2C9, STAT and JAK. We again manually searched related literature and further validated the biological relevance of the identified pathway segments. For example, HMOX1 often acts in concert with P450 cytochrome oxidoreductase (encoded by Por) and biliverdin reductase to convert heme into bilirubin, carbon monoxide and iron.^25^ HMOX1 expression has recently been shown to be regulated by interleukin-6 via the Jak/STAT pathway in hepatocytes.^26^

### 3.4. Comparison with other methods

As we discussed in the introduction section, several methods that have been developed to discover denovo pathways or pathway segments do not focus on conditionally activated pathway identification,^2^–^4^ and cannot output differentially activated pathway segments. Also, most of these methods are applied to yeast PPI data and are difficult to apply to larger networks. Ideker et al^27^ have developed a simulated annealing-based algorithm to identify differentially activated networks by utilizing differentially expressed genes and protein interaction networks. However, the method does not focus on TFs and pathway identification.

In addition, gene enrichment test methods such as GSEA^13^ have been developed for ranking the conditional relevance of a previously defined gene set, and therefore can only be applied to known pathways. We also applied GSEA to both breast cancer data and ET data. Table 5 listed the highest-ranked enriched pathways obtained by GSEA. Most of these previously defined pathways are not explicitly associated with the given phenotype, and none of them contain the activated TFs we have identified and which are supported by evidence in the literature.

## 4. Conclusions

In this paper, we developed a computational approach to detect conditionally activated TFs and their corresponding pathway segments from microarray and PPI data. Differing from current pathway analysis methods, the main features of our approach include two aspects. One is that we identify pathway segments by taking into account their corresponding experimental/physiological conditions. The other is that we consider downstream TF activation and incorporate TF activation information into pathway segment identification. We have applied our method to two microarray data sets and demonstrated the effectiveness of our approach by using literature search tools. With more information such as TF-target data and PPI data accumulated in the future, our approach will further assist in biological hypothesis generating and facilitate greater understanding of specific biological processes.

## Figures and Tables

**Table 1.** Activated TFs identified from breast cancer data.

**TFBS**	**p-value**	**q-value**	**TF**
CTTTGA_V$LEF1_Q2	5.89E-05	0.018	LEF1
V$FAC1_01	3.53E-05	0.018	FALZ
V$HFH3_01	2.67E-04	0.028	FOXI1(X)
V$PAX_Q6	2.77E-04	0.028	PAX5
V$OCT1_06	1.46E-04	0.028	POU2F1
CAGCTG_V$AP4_Q5	1.54E-03	0.041	REPIN1(X)
TTGTTT_V$FOXO4_01	9.53E-04	0.041	FOXO4
CAGGTG_V$E12_Q6	1.31E-03	0.041	ELSPBP1
V$MYOD_Q6_01	1.37E-03	0.041	MYOD
V$SP1_01	5.78E-04	0.041	SP1
V$FOXO1_01	1.18E-03	0.041	FOXO1
V$GATA2_01	1.52E-03	0.041	GATA2
V$IRF1_01	8.14E-04	0.041	IRF1
V$AREB6_03	6.92E-04	0.041	ZEB1

**Table 2.** Activated TFs identified from ET data.

**TFBS**	**p-value**	**q-value**	**TF**
V$HSF1_01	6.97E-06	0.004	HSF1
V$ATF4_Q2	2.31E-05	0.007	ATF4
V$RORA2_01	7.91E-05	0.012	RORA2
V$ERR1_Q2	0.00014	0.018	ESRRA
TGGAAA_V$NFAT_Q4_01	0.00036	0.028	NFAT5
V$GATA1_03	0.00043	0.028	GATA1
V$STAT_01	0.00042	0.028	STAT
V$RORA1_01	0.00049	0.028	RORA
V$YY1_Q6	6.00E-04	0.031	YY1
GGGAGGRR_V$MAZ_Q6	0.00065	0.031	MAZ
V$NFMUE1_Q6	0.00077	0.034	NFMUE1
V$CREB_Q4	0.00113	0.041	CREB1
V$PXR_Q2	0.00113	0.041	PXR
V$FOXJ2_02	0.00105	0.041	FOXJ2
V$CEBPB_02	0.00124	0.042	CEBPB
V$ZIC2_01	0.00136	0.042	ZIC2
V$USF_Q6	0.00134	0.042	USF
V$AML_Q6	0.00159	0.047	AML

**Table 3.** Relevance matrix corresponding to a breast cancer pathway segment (by PubMatrix tool).

**PubMatrix**	**Breast cancer**	**JUP**	**MUC1**	**ERBB4**	**ERBB3**	**ADAM17**
JUP	17	203	2	0	0	0
MUC1	533	2	2228	2	4	1
ERBB4	215	0	2	869	294	0
ERBB3	159	0	4	294	712	1
ADAM17	9	0	1	0	1	246

**Table 4.** Relevance matrix corresponding to an ET pathway segment (by PubMatrix tool).

**PubMatrix**	**CYP2C19**	**POR**	**HMOX1**	**BLVRB**	**CYP2C9**	**STAT**	**JAK2**	**JAK**	**Myeloproliferative disorder**
**CYP2C19**	1849	4	0	0	701	1	0	0	0
**POR**	4	2101	1	0	1	1	0	1	10
**HMOX1**	0	1	3746	0	1	18	1	5	5
**BLVRB**	0	0	0	2	0	0	0	0	0
**CYP2C9**	701	1	1	0	2093	1	0	0	1

**Table 5.** Top pathways obtained by GSEA method.

**Data set**	**Top three pathways obtained by GSEA method**
Breast cancer	Repression of pain sensation by the transcriptional regulator DREAM
	CARM1 and regulation of the Estrogen Receptor
	Circadian rhythm
ET	Cell Cycle: G2/M checkpoint
	Cell cycle pathway
	Role of EGF receptor trans-activation by GPCRs in cardiac hypertrophy
